# Supplementary material for: Psychosocial and Behavioral Effects of the COVID-19 Pandemic on Children and Adolescents with Autism and Their Families: Overview of the Literature and Initial Data from a Multinational Online Survey
Source: Healthcare (Basel). 2022 Apr 12;10(4):714. doi: 10.3390/healthcare10040714 (PMC9028372; doi:10.3390/healthcare10040714)
Supplement: Supplementary file 1 [file healthcare-10-00714-s001.zip › healthcare-1650632-supplementary.pdf]

File S1: Online Questionnaire (English)

This document contains supplemental material of the manuscript:

Kreysa, H., Schneider, D., Kowallik, A.E., Dastgheib, S.S., Doğdu, C., Kühn, G., Rutloff, J., & Schweinberger S.R. (2022). **Psychosocial and Behavioral Effects of the COVID-19 Pandemic on Children and Adolescents with Autism and their Families: Overview of the Literature and Initial Data from a Multinational Online Survey**.

CoronaAutism -. English 07.02.2021, 21:47

Page 01

01

Thank you very much for participating in our survey. The information you provide will help to provide insights into how the current pandemic has affected the daily life and well-being of families with children with autism in different countries.

Please note that you must first read through some ethical guidelines before the actual questionnaire begins.

Also note that there is no "Back" button. Therefore, please read each page carefully before proceeding to the next page and do not use the "Back" function in your Internet browser. Thank you very much.

Page 02

02

GR02

Information for Participants

Study: "Effects of the coronavirus pandemic on families and individuals with autism spectrum disorders"

By clicking "Next" you confirm that you have read the detailed information about the study that can be downloaded here.

VERY IMPORTANT: For most browser-settings the following applies when clicking on the download link: right click>open in the new tab. Otherwise you will not be able to continue with the questionnaire.

Page 03
03

GR03

Declaration of consent

I was informed in writing about the nature, significance, scope and risks of the scientific study and had the opportunity to clarify my questions about it with the study managers. I was presented with the "Information for Participants" of the study and provided with a download link for this information.

I agree to participate **in** the study. My participation is voluntary.

I have the right to end my participation at any time without giving reasons and without any negative consequences for the future.

I accept that the personal data collected in the course of this scientific study:

- will at no time be linked to my real name, place of residence or e-mail address,
- be used exclusively for scientific research,
- will initially only be accessible to the scientists involved in this study (Prof. Dr. Stefan Schweinberger, Dr. Dana Schne ider, Dr. Helene Kreysa, Samaneh Dastgheib, Andrea Kowallik and Cem Dogdu) and other members of the research group "Social Potential in Autism" at the Friedrich Schiller University of Jena. For possible continuation or replication work, the data can be made available to other researchers in completely anonymous form,
- will be stored, processed and analysed on electronic data carriers and the results obtained will be published in anonymous form.

By clicking "Next", you confirm your consent.

Page 04
co

GR20

Please create an anonymous code.

The code consists of the first two letters of your mother's first name, the day you were born, and the last two letters of your own first name.

Example:

Mother= Monica

Your birthday= October 21st

Your first name = John

Code: M021HN

Please write your personal code in the blank text field.

GR19

Page 05

04

By beginning the questionnaire, you confirm that you are the care-giver for a sch5 age child with a clinical diagnosis on the Autism Spectrum, living in the same *household.*

Important; *In the following questionnaire, we will refer to this child with* **Autism** as ***Child A.*** *We will* also ask *you to fill out* some *questions about one other child* ***(Child BJ*** *who ideally lives in the* same *household. If you do not have another child in your household please report about another child with whom you are in close contact. We will refer to this child* as ***Child B.*** *Child B should* ***not*** *have a clinical diagnosis on the Autism Spectrum.*

Please note that not all questions in this survey may cover all aspects that were important to you. Therefore, you will find a box for open comments at the end.

Page 06

8

In this section, we ask you to provide us with some current information ab6 l yourself, your children, where you live, and how the pandemic has affected you.

SIOl

How many people (including yourself) currently live in your household most of the time?

e 1
e 2
e 3
e 4
e 5
e 6
e 7
e a
e g
e m
e more than 10

5D19

For each household member (beginning with yourself) please provide some basic information.

GR26

I am giving detailed information about...

5D20

- e Myself
- • Child A- with Autism
- e Child 8- without Autism
- e Other sibling
- e Other parent
- e Other household member

SIOS

Age in full years

SA01

Gender

The person is...

- e female
- e male
- e other
- e prefer not to say

SA04

What is the profession of this person?

- Pupil/in school
- Training/apprenticeship
- University student
- Employee- Full time
- Employee- Part time
- Civil servant
- Self-employed
- Unemployed/seeking employment
- Other

B137

Is a clinical diagnosis on the Autism Spectrum known for this person?

- Yes
- Suspected
- No

GR27

I am giving detailed information about...

5D49

- Myself
- Child A- with Autism
- Child B- without Autism
- Other sibling
- Other parent
- Other household member

SA83

Age in full years

SA02

Gender

The person is...

- female
- male
- other
- prefer not to say

SA38

What is the profession of this person?

- Pupil/in school
- Training/apprenticeship
- University student
- Employee- Full time
- Employee- Part time
- Civil servant
- Self-employed
- Unemployed/seeking employment

Other

B138

Is a clinical diagnosis on the Autism Spectrum known for this person?

- e Yes
- e Suspected
- • No

GR28

I am giving detailed information about...

5D53

- e Myself
- • Child A- with Autism
- • Child B- without Autism
- e Other sibling
- e Other parent
- e Other household member

SA87

Age in full years

SA03

Gender

The person is...

- e female
- e male
- e other
- e prefer not to say

SA39

What is the profession of this person?

- Pupil/in school
- Training/apprenticeship
- University student
- Employee- Full time
- Employee- Part time
- Civil servant
- Self-employed
- Unemployed/seeking employment
- Other

B28

Is a clinical diagnosis on the Autism Spectrum known for this person?

- Yes
- Suspected
- No

GR29

I am giving detailed information about...

5D54

- Myself
- Child A- with Autism
- Child B- without Autism
- Other sibling
- Other parent
- Other household member

SA91

Age in full years

SA16

Gender

The person is...

- female
- male
- other
- prefer not to say

SA40

What is the profession of this person?

- Pupil/in school
- Training/apprenticeship
- University student
- Employee- Full time
- Employee- Part time
- Civil servant
- Self-employed
- Unemployed/seeking employment
- Other

B29

Is a clinical diagnosis on the Autism Spectrum known for this person?

e Yes
e Suspected
• No

GR30

I am giving detailed information about...

SDSS

e Myself
• Child A- with Autism
• Child B- without Autism
e Other sibling
e Other parent
e Other household member

SA95

Age in full years

SA17

Gender

The person is...

e female
e male
e other
e prefer not to say

SA41

What is the profession of this person?

- Pupil/in school
- Training/apprenticeship
- University student
- Employee- Full time
- Employee- Part time
- Civil servant
- Self-employed
- Unemployed/seeking employment

Other

B30

Is a clinical diagnosis on the Autism Spectrum known for this person?

- Yes
- Suspected
- No

GR46

I am giving detailed information about...

5D56

- Myself
- Child A- with Autism
- Child B- without Autism
- Other sibling
- Other parent
- Other household member

SA99

Age in full years

SA18

Gender

The person is...

- female
- male
- other
- prefer not to say

SA42

What is the profession of this person?

- Pupil/in school
- Training/apprenticeship
- University student
- Employee- Full time
- Employee- Part time
- Civil servant
- Self-employed
- Unemployed/seeking employment
- Other

B31

Is a clinical diagnosis on the Autism Spectrum known for this person?

- e Yes
- e Suspected
- • No

Page 08

Diagnosis

B129

For Child A, please indicate the severity of impairment due to their Autism Spectrum condition.

- e requires little or no support
- e requires support
- e requires substantial support
- e requires very substantial support

B130 1!!1

Is Child A impaired in any other way?

- ● yes (we would be very grateful if you could provide further information)
- e no
- e prefer not to say

Page 09

06

Please indicate how much the following questions apply to Child A. Please tick one option per question 'a?..?J.

	strongly agree	agree	disagree	strongly disagree
1. S/he often notices small sounds when others do not.				
2. S/he usually concentrates more on the whole picture, rather than the small details.				
3. In a social group, s/he can easily keep track of several different people's conversations.				
4. S/he finds it easy to go back and forth between different activities.				
5. S/he doesn't know how to keep a conversation going with his/her peers.				
6. S/he is good at social chit-chat.				
7. Whens/he is read a story, s/he finds it difficult to work out the character's intentions or feelings.				
8. When s/he was in preschool, s/he used to enjoy playing games involving pretending with other children				
9. S/he finds it easy to work out what someone is thinking or feeling just by looking at their face				
10. S/he finds it hard to make new friends				

Please indicate how much the following questions apply to Child B. Please tick one option per question 'a?..?l

	strongly agree	agree	disagree	strongly disagree
1. S/he often notices small sounds when others do not.				
2. S/he usually concentrates more on the whole picture, rather than the small details.				
3. In a social group, s/he can easily keep track of several different people's conversations				
4. S/he finds it easy to go back and forth between different activities.				
5. S/he doesn't know how to keep a conversation going with his/her peers.				
6. S/he is good at social chit-chat.				
7. Whens/he is read a story, s/he finds it difficult to work out the character's intentions or feelings.				
8. When s/he was in preschool, s/he used to enjoy playing games involving pretending with other children				
9. S/he finds it easy to work out what someone is thinking or feeling just by looking at their face				
10. S/he finds it hard to make new friends				

Page 10
07

What kind of accommodation do you live in? D501

- flat
- house
- other ______

Do any household members live somewhere else some of the time? D502

- No
- Yes, please specify ______

Does your accommodation have direct access to the outside? D503

- no
- balcony
- garden
- yard
- other, please specify ______

How many bedrooms are there in your accommodation? D504

- 0
- 1
- 2
- 4
- 5
- 6
- 7
- a
- 9
- 10 or more

Is there a private room for each child in the household? D518

- yes
- no
- unclear

Page 11

08

DC01

Please indicate the population size of your community.

- e less than one hundred
- e between one hundred and one thousand
- e between one thousand and ten thousand
- e between ten thousand and one hundred thousand
- e between one hundred thousand and one million
- e more than one million

EDOS

Which is the country, you're currently living in?

Country:

ED17

Were you born where you are currently living?

- e yes, in this country and in this community/ town
- e yes, in this country, but in a different community/ town
- e in a different country
- e prefer not to say

We are planning to circulate this survey in several different countries, so pl se let us know about the situation where you live.

Where you live, did the current pandemic lead to the following administrative restrictions at any time? CP15

School closures

Complete lockdown

Where you live, are the following measures currently still in place? CP02

	Yes	No	Partially	I don' t know
School closures	●	●	●	●
Complete lockdown	●	●	●	●
Limits on groups of people	●	●	●	●
Limits on time and activities outside the house	●	●	●	●
Wearing protective clothing / face masks in public	●	●	●	●
Travel restrictions	●	●	●	●
Limits on sports and cultural activities	●	●	●	●
Restrictions for religious events	●	●	●	●
Restricted (non-corona) health-care and therapy options	●	●	●	●
Mandatory quarantine	●	●	●	●
Other (e.g., requirement/ encouragement to carry tracking devices, documentation of purpose of visit or health status, ,,,)	●	●	●	●

What is your personal prediction about the future regarding the current crisis? When do you think the pf 8lmic will be over?

- e 1 month
- e 3 months
- e 6 months
- e 1 year
- e several years
- e never

Page 13

10 A

AC01

In the following, we are interested in how children and adolescents on the Autism spectrum spend their day.

For each of the following activities, please estimate how much time Child A (i.e., the autistic person you ?i s reporting about) spent / spends on it in hours on a typical 24-hour day. Note, you can give proportions of the hour (e.g., 30 min = 0.5).

1. Use of digital devices (smartphone, tablet, laptop) for social communication (e.g. chatting with friendlf a family).

Before corona restrictions

[Please choose] v

ACSS

At the peak of the crisis

[Please choose] v

AC59

Currently

[Please choose] v

2. Use of digital devices (smartphone, tablet, laptop) for entertainment (e.g., games, films, surfing the W[illegible]",

Before corona restrictions

[Please choose] v

AC60

At the peak of the crisis

[Please choose] v

AC68

Currently

[Please choose] v

AA79

3. Use of digital devices (smartphone, tablet, laptop) for education (e.g., homeschooling).

Before corona restrictions

[Please choose] v

AA78

At the peak of the crisis

[Please choose] v

Currently

AA77

[Please choose] v

4. Use of digital devices (smartphone, tablet, laptop) for - therapy, consultation, and coaching.

AA72

Before corona restrictions

[Please choose] v

At the peak of the crisis

AA71

[Please choose] v

Currently

AA89

[Please choose] v

5. Special interests and hobbies.

AA66

Before corona restrictions

[Please choose] v

At the peak of the crisis

AA65

[Please choose] v

Currently

AA64

[Please choose] v

6. Inside the house.

AD01

Before corona restrictions

[Please choose] v

At the peak of the crisis

AD02

[Please choose] v

Currently

AD03

[Please choose] v

AD07

7. In the garden or on the balcony.

Before corona restrictions

[Please choose] v

ADOS

At the peak of the crisis

[Please choose] v

AD09

Currently

[Please choose] v

AD13

8. Outside (but not in your own garden or on your own balcony).

Before corona restrictions

[Please choose] v

AD14

At the peak of the crisis

[Please choose] v

AD15

Currently

[Please choose] v

AD19

9. Sleeping or in bed.

Before corona restrictions

[Please choose] v

AD20

At the peak of the crisis

[Please choose] v

AD21

Currently

[Please choose] v

Page 14

Activities

In the following, we are interested in how other children and adolescents sp[illegible]d their day.

For each of the following activities, please now estimate how much time Child B (i.e., the other child/ado[illegible] you are reporting about) spent / spends on it in hours on a typical 24-hour day. Note again, you can give proportions of the hour (e.g., 30 min = 0.5).

1. Use of digital devices (smartphone, tablet, laptop) for social communication (e.g. chatting with frien[illegible] family).

Before corona restrictions

[Please choose] v

AC90

At the peak of the crisis

[Please choose] v

AC91

Currently

[Please choose] v

[illegible]

2. Use of digital devices (smartphone, tablet, laptop) for entertainment (e.g., games, films, surfing the W[illegible],

Before corona restrictions

[Please choose] v

AC96

At the peak of the crisis

[Please choose] v

AC97

Currently

[Please choose] v

AA81

3. Use of digital devices (smartphone, tablet, laptop) for education (e.g., homeschooling).

Before corona restrictions

[Please choose] v

AA82

At the peak of the crisis

[Please choose] v

Currently

AA83

[Please choose] v

AA87

4. Use of digital devices (smartphone, tablet, laptop) for - therapy, consultation, and coaching.

Before corona restrictions

[Please choose] v

AA88

At the peak of the crisis

[Please choose] v

AA70

Currently

[Please choose] v

AA93

5. Special interests and hobbies.

Before corona restrictions

[Please choose] v

AA94

At the peak of the crisis

[Please choose] v

AA95

Currently

[Please choose] v

AD25

6. Inside the house.

Before corona restrictions

[Please choose] v

AD26

At the peak of the crisis

[Please choose] v

AD27

Currently

[Please choose] v

AD31

7. In the garden or on the balcony.

Before corona restrictions

[Please choose] v

AD32

At the peak of the crisis

[Please choose] v

AD33

Currently

[Please choose] v

AD37

8. Outside (but not in your own garden or on your own balcony).

Before corona restrictions

[Please choose] v

AD38

At the peak of the crisis

[Please choose] v

AD39

Currently

[Please choose] v

AD43

9. Sleeping or in bed.

Before corona restrictions

[Please choose] v

AD44

At the peak of the crisis

[Please choose] v

AD45

Currently

[Please choose] v

Page 15

HR

HB01

The next questions concern changes in the health and well-being of children and adolescents.

Please rate the following aspects for Child A at the present moment in time, compared to the time beforeHffl>J current pandemic.

	much decreased	slightly decreased	similar	slightly increased	much increased
General well-being	●	●	●	●	●
Adaptation abilities	●	●	●	●	●
Overall anxiety levels	●	●	●	●	●
Emotional reactions	●	●	●	●	●
Social behaviour	●	●	●	●	●

Please rate the following aspects for Child B at the present moment in time, compared to the time beforeHffl>J current pandemic.

	much decreased	slightly decreased	similar	slightly increased	much increased
General well-being	●	●	●	●	●
Adaptation abilities	●	●	●	●	●
Overall anxiety levels	●	●	●	●	●
Emotional reactions	●	●	●	●	●
Social behaviour	●	●	●	●	●

Please indicate how Child A responds to the following pandemic hygiene routines. HBOS

	not applicable	negative	neutral	positive
Maintaining physical distance to other people (1.5 m)	●	●	●	●
Regular hand-washing	●	●	●	●
Use of disinfectant	●	●	●	●
Wearing a face mask	●	●	●	●

Please indicate how Child B responds to the following pandemic hygiene routines. HB07

	not applicable	negative	neutral	positive
Maintaining physical distance to other people (1.5 m)	●	●	●	●
Regular hand-washing	●	●	●	●
Use of disinfectant	●	●	●	●
Wearing a face mask	●	●	●	●

Page 18
12

PP80

PedsQL™

Pediatric Quality of Life Inventory™

Version 4.0

Parent Report for Children (ages 8-12)

PedsQL™ Copyright © 1998 JW Varni, Ph.D. All rights reserved.

PP53

All originals items of the PedSql are copyrighted. Therefore, we have only presented the parts that reflect our own instructions specific to this study.

PQ49

You are now filling this in for Child A.

PQ02

Items related to the problems with physical functioning.

1.

- 0-
- 1-
- 2-
- 3-
- 4-

PQ66

2.

- 0-
- 1-
- 2-
- 3-
- 4-

3. Participating in sports activity or exercise

PQ67

e 0- Never
e 1- Almost Never
e 2- Sometimes
e 3- Often
e 4- Almost Always

PI25

4. Lifting something heavy

e 0- Never
e 1- Almost Never
e 2- Sometimes
e 3- Often
e 4- Almost Always

PI27

5. Taking a bath or shower by him or herself

e 0- Never
e 1- Almost Never
e 2- Sometimes
e 3- Often
e 4- Almost Always

PI29

6. Doing chores around the house

e 0- Never
e 1- Almost Never
e 2- Sometimes
e 3- Often
e 4- Almost Always

PI31

7. Having hurts and aches

e 0- Never
e 1- Almost Never
e 2- Sometimes
e 3- Often
e 4- Almost Always

PI33

8. Low energy level

- e 0- Never
- e 1- Almost Never
- e 2- Sometimes
- e 3- Often
- e 4- Almost Always

PP88

PedsQLTM

Pediatric Quality of Life InventoryTM

Version 4.0

Parent Report for Children (ages 8-12)

PedsQLTM Copyright© 1998 JW Varni, Ph.D. All rights reserved.

PI65

In Items related to the problems with emotional functioning: **your child had with...**

EMOTIONAL FUNCTIONING (problems with...)

1. Feeling afraid or scared

- 0- Never
- 1- Almost Never
- 2- Sometimes
- 3- Often
- 4- Almost Always

PI66

2. Feeling sad or blue

- 0- Never
- 1- Almost Never
- 2- Sometimes
- 3- Often
- 4- Almost Always

PI67

3. Feeling angry

- 0- Never
- 1- Almost Never
- 2- Sometimes
- 3- Often
- 4- Almost Always

PI68

4. Trouble sleeping

e 0- Never
e 1- Almost Never
e 2- Sometimes
e 3- Often
e 4- Almost Always

PT01

5. Worrying about what will happen to him or her

e 0- Never
e 1- Almost Never
e 2- Sometimes
e 3- Often
e 4- Almost Always

PP89

PedsQLTM

Pediatric Quality of Life InventoryTM

Version 4.0

Parent Report for Children (ages 8-12)

PedsQLTM Copyright© 1998 JW Varni, Ph.D. All rights reserved.

PT25

In Items related to the problems with social functioning: **has your child had with...**

SOCIAL FUNCTIONING (problems with...)

1. Getting along with other children

- e 0- Never
- e 1- Almost Never
- e 2- Sometimes
- e 3- Often
- e 4- Almost Always

PT26

2. Other kids not wanting to be his or her friend

- e 0- Never
- e 1- Almost Never
- e 2- Sometimes
- e 3- Often
- e 4- Almost Always

PT27

3. Getting teased by other children

- e 0- Never
- e 1- Almost Never
- e 2- Sometimes
- e 3- Often
- e 4- Almost Always

PT28

4. Not able to do things that other children his or her age can do

- e 0- Never
- e 1- Almost Never
- e 2- Sometimes
- e 3- Often
- e 4- Almost Always

PT29

5. Keeping up when playing with other children

- e 0- Never
- e 1- Almost Never
- e 2- Sometimes
- e 3- Often
- e 4- Almost Always

PP09

Items related to the problems with school functioning:

In the past ONE month, how much of a problem has your child had with...

SCHOOL FUNCTIONING (problems with...)

1. Pay attention in class

- e 0- Never
- e 1- Almost Never
- e 2- Sometimes
- e 3- Often
- e 4- Almost Always

PP10

2. Forgetting things

- e 0- Never
- e 1- Almost Never
- e 2- Sometimes
- e 3- Often
- e 4- Almost Always

PP11

3. Keeping up with schoolwork

- e 0- Never
- e 1- Almost Never
- e 2- Sometimes
- e 3- Often
- e 4- Almost Always

PP12

4. Missing schoo because of not feeling well

- e 0- Never
- e 1- Almost Never
- e 2- Sometimes
- e 3- Often
- e 4- Almost Always

PP13

5. Missing school to go to the doctor or hospital

- e 0- Never
- e 1- Almost Never
- e 2- Sometimes
- e 3- Often
- e 4- Almost Always

PP49

For any information on the use of the **PedsQLTM** , please contact Mapi Research Trust, Lyon, France.

Internet: https://eprovide.mapi-trust.org

PP84

PedsQLTM

Pediatric Quality of Life InventoryTM

Version 4.0

Parent Report for Children (ages 8-12)

PedsQL TM Copyright© 1998 JW Varni, Ph.D. All rights reserved.

PPS4

DIRECTIONS

On the following screens are things that might be a problem for your child.

Please tell us how much of a problem each one has been for your child during the past ONE month by selecting:

0 if it is never a problem

1 if it is almost never a problem

2 if it is sometimes a problem

3 if it is often a problem

4 if it is almost always a problem

There are no right or wrong answers. If you do not understand a question, please ask for help.

PQSO

You are now filling this in for Child B.

PQ73

Items related to the problems with physical functioning.

In the past ONE month, how much of a problem has your child had with...

PHYS CAL FUNCTION NG (problems with...)

1. Walking more than one block

e 0- Never
e 1-Almost Never
e 2- Sometimes
e 3- Often
e 4- Almost Always

PQ74

2. Running

e 0- Never
e 1-Almost Never
e 2- Sometimes
e 3- Often
e 4- Almost Always

3. Participating in sports activity or exercise

PQ7S

- 0- Never
- 1- Almost Never
- 2- Sometimes
- 3- Often
- 4- Almost Always

PI26

4. Lifting something heavy

- 0- Never
- 1- Almost Never
- 2- Sometimes
- 3- Often
- 4- Almost Always

PI28

5. Taking a bath or shower by him or herself

- 0- Never
- 1- Almost Never
- 2- Sometimes
- 3- Often
- 4- Almost Always

PI30

6. Doing chores around the house

- 0- Never
- 1- Almost Never
- 2- Sometimes
- 3- Often
- 4- Almost Always

PI32

7. Having hurts and aches

- 0- Never
- 1- Almost Never
- 2- Sometimes
- 3- Often
- 4- Almost Always

PI34

8. Low energy level

- e 0- Never
- e 1- Almost Never
- e 2- Sometimes
- e 3- Often
- e 4- Almost Always

PP96

PedsQLTM

Pediatric Quality of Life InventoryTM

Version 4.0

Parent Report for Children (ages 8-12)

PedsQL TM Copyright© 1998 JW Varni, Ph.D. All rights reserved.

Items related to the problems with emotional functioning:

P170

In the past ONE month, how much of a problem has your child had with...

Ef:>TIONAL FUNCTIONING (problems with...)

1. **leeling** afraid or scared

e 0- Never
e 1- Almost Never
e 2- Sometimes
e 3- Often
e 4- Almost Always

Pl71

2. Feeling sad or blue

e 0- Never
e 1- Almost Never
e 2- Sometimes
e 3- Often
e 4- Almost Always

Pl72

3. Feeling angry

e 0- Never
e 1- Almost Never
e 2- Sometimes
e 3- Often
e 4- Almost Always

Pl73

4. Trouble sleeping

- e 0- Never
- e 1- Almost Never
- e 2- Sometimes
- e 3- Often
- e 4- Almost Always

PT03

5. Worrying about what will happen to him or her

- e 0- Never
- e 1- Almost Never
- e 2- Sometimes
- e 3- Often
- e 4- Almost Always

PP97

PedsQLTM

Pediatric Quality of Life InventoryTM

Version 4.0

Parent Report for Children (ages 8-12)

PedsQL TM Copyright© 1998 JW Varni, Ph.D. All rights reserved.

Items related to the problems with social functioning:

In the past ONE month, how much of a problem has your child had with...

PT30

SOCIAL FUNCTIONING (problems with...)

1. Getting along with other children

e 0- Never
e 1- Almost Never
e 2- Sometimes
e 3- Often
e 4- Almost Always

PT31

2. Other kids not wanting to be his or her friend

e 0- Never
e 1- Almost Never
e 2- Sometimes
e 3- Often
e 4- Almost Always

PT32

3. Getting teased by other children

e 0- Never
e 1- Almost Never
e 2- Sometimes
e 3- Often
e 4- Almost Always

PT33

4. Not able to do things that other children his or her age can do

- e 0- Never
- e 1- Almost Never
- e 2- Sometimes
- e 3- Often
- e 4- Almost Always

PT34

5. Keeping up when playing with other children

- e 0- Never
- e 1- Almost Never
- e 2- Sometimes
- e 3- Often
- e 4- Almost Always

PP14

In Items related to the problems with school functioning: **has your child had with...**

SCHOOL FUNCTIONING (problems with...)

1. Pay attention in class

- e 0- Never
- e 1- Almost Never
- e 2- Sometimes
- e 3- Often
- e 4- Almost Always

PP15

2. Forgetting things

- e 0- Never
- e 1- Almost Never
- e 2- Sometimes
- e 3- Often
- e 4- Almost Always

PP16

3. Keeping up with schoolwork

- e 0- Never
- e 1- Almost Never
- e 2- Sometimes
- e 3- Often
- e 4- Almost Always

PP17

4. Missing school because of not feeling well

- 0- Never
- 1- Almost Never
- 2- Sometimes
- 3- Often
- 4- Almost Always

PP18

5. Missing school to go to the doctor or hospital

- 0- Never
- 1- Almost Never
- 2- Sometimes
- 3- Often
- 4- Almost Always

PPSS

For any information on the use of the **PedsQLTM** , please contact Mapi Research Trust, Lyon, France.

Internet: https://eprovide.mapi-trust.org

Page 24

13

1

Now please let us know how the current pandemic affected your entire famy or household.

Please rate the following aspects for your family at the present moment in time, compared to the time beVoii the current pandemic.

	much decreased	slightly decreased	similar	slighlty increased	much increased
Overall stress levels	●	●	●	●	●
Regular daily structure	●	●	●	●	●
Conflict	●	●	●	●	●
Time spent together	●	●	●	●	●
Emotional closeness	●	●	●	●	●

Page 25

14

During the current pandemic did you receive any form of support through the following people or institufi'J>Js? Please indicate all that apply.

Support by....

- Close family
- Extended family and friends
- Neighbours
- Institutions
- Health service
- Government

ST02

Which (if any) forms of support did you find particularly useful and why?

Close family, because...

STOS

Extended family and friends, because...

ST06

Neighbours, because...

ST07

Institutions, because ...

STOS

Health service, because...

ST09

Government, because...

ST01

(How) did the current pandemic affect your child's ASD-specific educational and therapy/ rehabilitation activities?

Did you experience any other specific challenges during the current pandemic? ST04

Page 26

OQ

This is the open answer box that allows you to tell us about other aspects Jt3t'1e pandemic that were important to you.

ST42

Last Page

Thank you for completing this questionnaire!

We would like to thank you very much for helping us.

Your answers were transmitted, you may close the browser window or tab now.

Dr. Dana Schneider, Friedrich Schiller University Jena - 2020
